# Randomised, controlled Trial of CT perfusion and angiography compared to CT alone in thrombolysis-eligible acute ischaemic stroke patients: The penumbra and recanalisation acute computed tomography in ischaemic stroke evaluation (PRACTISE) trial

**DOI:** 10.1093/esj/23969873251372348

**Published:** 2026-01-01

**Authors:** Keith W Muir, Salwa El Tawil, Alex McConnachie, Ian Ford, Grant Mair, Jattinder Khaira, Kausik Chatterjee, Laszlo Sztriha, Omid Halse, Ibrahim Balogun, Sanjeev Nayak, Phil White, Elizabeth A Warburton, Joanna Wardlaw

**Affiliations:** School of Cardiovascular & Metabolic Health, University of Glasgow, Queen Elizabeth University Hospital, Glasgow, Scotland, UK; School of Cardiovascular & Metabolic Health, University of Glasgow, Queen Elizabeth University Hospital, Glasgow, Scotland, UK; Robertson Centre for Biostatistics, School of Health and Wellbeing, University of Glasgow, Glasgow, Scotland, UK; Robertson Centre for Biostatistics, School of Health and Wellbeing, University of Glasgow, Glasgow, Scotland, UK; Centre for Clinical Brain Sciences and Edinburgh Imaging, University of Edinburgh, Edinburgh, UK; University Hospitals Birmingham, Birmingham, England UK; Countess of Chester Hospital, Chester, Cheshire West, Chester, UK; King’s College Hospital, London, UK; Charing Cross Hospital, London, UK; Kent and Canterbury Hospital, Canterbury, Ashford, Kent, UK; Royal Stoke University Hospital, Stoke-on-Trent, Staffordshire, UK; Translational and Clinical Research Institute, Newcastle University, Newcastle upon Tyne, UK; University of Cambridge, Cambridge, Cambridgeshire, UK; Centre for Clinical Brain Sciences and Edinburgh Imaging, University of Edinburgh, Edinburgh, UK; UK Dementia Research Institute Centre at the University of Edinburgh, Edinburgh, UK

**Keywords:** Stroke, ischaemic stroke, imaging, computed tomography, perfusion, angiography, thrombolysis

## Abstract

**Introduction:**

The role of CT angiography (CTA) and CT perfusion (CTP) in patient selection for thrombolysis <4.5 h after onset is unclear. Additional imaging may improve specificity of diagnosis by excluding stroke mimics or those without salvageable tissue, but may delay treatment.

**Patients and methods:**

In a multicentre prospective randomised trial, thrombolysis-eligible patients <4.5 h from symptom onset were randomised 1:1 to non-contrast CT (NCCT) or multimodal CT (NCCT + CTA + CTP). The primary endpoint was the proportion receiving thrombolysis. Secondary end-points were times to decision-making and treatment delivery, early neurological recovery, functional recovery at 3 months and incidence of symptomatic intracerebral haemorrhage (SICH).

**Results:**

Between March 2015 and May 2018, 271 patients were randomised, 134 to multimodal CT and 137 to NCCT. After initial NCCT, 114 had no contraindication to thrombolysis in the multimodal CT group and 108 in the NCCT group. Mean age was 67.5 years and median NIHSS score was 6 (interquartile range 3–12). Fewer patients assigned multimodal CT received thrombolysis (56/114, 49.1%) compared to NCCT (73/108, 67.6%, adjusted odds ratio (aOR) 0.46 (95% CI: 0.25–0.83), *p* = 0.0102). Times to treatment decision or thrombolytic administration, early neurological recovery and day 90 functional outcome did not differ significantly. SICH occurred in two patients, both assigned NCCT. Mortality was 6/114 (5.3%) in the multimodal CT group compared to 11/108 (10.2%; aOR 0.46 (95% CI: 0.16, 1.31), *p* = 0.147) in the NCCT group.

**Discussion:**

Despite fewer patients receiving thrombolysis after multimodal imaging, treatment decision times and clinical outcomes did not differ significantly. Multimodal CT may identify patients who do not require thrombolysis such as stroke mimics and non-disabling strokes.

**Conclusion:**

Among acute stroke patients imaged <4.5 h from symptom onset, multimodal CT reduced use of thrombolysis. Treatment decision times and clinical outcomes did not differ between groups.

## Graphical Abstract

**Graphical abstract:**
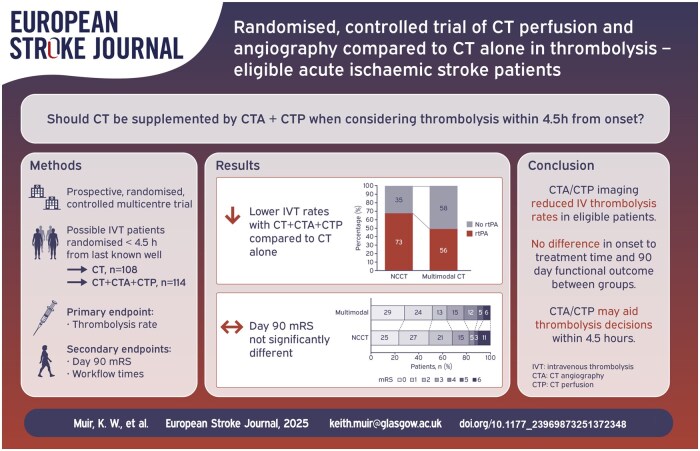

## Introduction

Almost all clinical trials of intravenous thrombolysis within 4.5 h of onset of acute ischaemic stroke used non-contrast-enhanced computed tomography (NCCT) for brain imaging since NCCT is fast, widely available, and highly sensitive for identification of acute intracranial haemorrhage, the main contraindication to treatment.^1^ The sensitivity of NCCT to acute ischaemia is limited, however, particularly with earlier imaging^2^ or among those with less severe symptoms^3^; even for moderate to severe stroke within the first 6 h after symptom onset, NCCT sensitivity is only 60%.^4^ Since structural non-stroke pathology on imaging is infrequently observed, treatment decisions are commonly based on clinical judgement and absence of haemorrhage. Clinician uncertainty about risk-benefit balance, coupled with time pressure contributed historically to under-utilisation of thrombolysis, particularly in some patient groups such as those with mild or improving symptoms.^5^ Recent evidence suggests a shift in clinical behaviour with increasing thrombolysis treatment rates^5,6^ but also a high number of patients with non-stroke diagnoses (stroke mimics) being given thrombolytic drugs in both clinical trials^7,8^ and in routine practice.^9^

CT angiography (CTA) and CT perfusion (CTP) substantially increase diagnostic sensitivity compared to NCCT and additionally characterise the severity, location and extent of brain ischaemia^3^ but are implemented to a variable extent in clinical practice. CTA is required to diagnose large vessel occlusion (LVO) suitable for endovascular thrombectomy, and CTP has been used predominantly to select patients for thrombolysis or thrombectomy when onset time is unknown or in late time windows (>4.5 or 6 h after onset).^10,11^

The value of additional CTP among patients presenting <4.5 h after onset, or of CTA in less severely affected patients, is uncertain. Increased diagnostic sensitivity may increase treatment of patients currently excluded on grounds of clinical uncertainty. It may also increase the specificity of treatment by reducing inappropriate thrombolytic administration in stroke mimics, or to patients with minor stroke in whom benefit is uncertain.^8^ Alternatively, additional imaging may delay treatment or reduce likelihood of treatment in patients lacking obvious arterial occlusion or perfusion defects, and it increases the radiation dose received by patients. In experienced centres, combined NCCT, CTA and CTP (multimodal CT) for patient selection for intravenous thrombolysis appeared feasible and safe, without major delays in door-to-needle times, but generalisability and impact on outcome is uncertain,^3,12^ given the absence of randomised evidence of the effect of multimodal versus plain CT imaging. Although IST-3 did not show better outcomes in 141/3032 participants randomised to alteplase versus no alteplase with CTP versus only NCCT, the use of imaging was not randomised.^13^

We undertook a randomised, controlled clinical trial to compare the impact of multimodal CT imaging (NCCT and additional CTA and CTP) with NCCT alone on treatment rates with intravenous thrombolysis in acute ischaemic stroke.^14^

## Patients and methods

### Study design

We undertook a multicentre prospective randomised trial. Functional endpoints were assessed blind to imaging strategy allocation. The trial was approved by multicentre Research Ethics Committees (REC reference numbers: Scotland REC 14/SS/0113, England REC 14/EM/1291). The protocol has been published^14^ and the trial was registered (ClinicalTrials.gov identifier NCT02360670). Participating sites were established thrombolysis centres participating in national audits that had prior experience with multimodal imaging. We recruited adult patients with clinically diagnosed acute stroke <4.5 h after symptom onset who were considered potentially eligible for treatment with intravenous thrombolysis. Patients were excluded if they had known contraindications to thrombolytic drug treatment, significantly impaired renal function, allergy to iodinated contrast or any intercurrent medical condition that would prevent participation in study procedures or with life expectancy ⩽3 months.

### Randomisation and masking

After consent from patients or their legal representatives, patients were randomised via an interactive voice response system to either routine imaging (NCCT alone) or multimodal CT imaging (NCCT + CTA + CTP) in a 1:1 ratio. A minimisation algorithm, with a small random element, was used to ensure balance between randomised groups in terms of study site, stroke severity (NIHSS 0–6, 7–16, 17–25), and hemispheric lateralisation (right, left, bilateral). If initial NCCT identified contraindications to thrombolysis (intracranial haemorrhage, non-stroke abnormalities potentially responsible for the presentation such as tumour, or extensive established stroke), the patient was regarded as a screen failure and had no further involvement in the study. When NCCT confirmed no imaging contraindications to thrombolytic treatment, those randomised to multimodal CT proceeded to additional CTA and CTP as soon as possible, ideally immediately after NCCT.

### Study assessments

Site staff collected baseline clinical data including demographics, medical history, stroke severity assessed by the National Institutes of Health Stroke Scale (NIHSS) score and estimated pre-stroke disability by the modified Rankin Scale (mRS).

The order of scan acquisition was at the discretion of each centre. CTA covering aortic arch to vertex was recommended. Suggested CTP parameters included 60-s acquisition time, maximal *z*-axis coverage and slice thickness ⩽5 mm. Sites were permitted to use whatever software they wished for processing and review. Choice of CTP parameter maps was at the discretion of each site. Local review of CTA and CTP was used to inform decisions at the discretion of the clinical team. No specific criteria for treatment were recommended by the trial protocol; sites were instructed to consider treatment as per UK guidelines current at the time.^15^ Local imaging interpretation was documented, including presence and location of CTA occlusion, NCCT findings and CTP perfusion patterns including the estimated extent of core-penumbra mismatch. Thrombectomy was permitted if clinically indicated and locally available. Times of symptom onset, hospital arrival, treatment decision and start time for thrombolytic drug treatment if given, were recorded. At 24 h repeat NCCT and NIHSS were undertaken. All patients were followed up for clinical outcomes (early neurological change measured by NIHSS and complications up to day 7 or hospital discharge if earlier by site staff), and by central blinded staff at telephone interview at day 90 to determine functional status on the modified Rankin Score (mRS).

Central review of NCCT and CTA was managed by the University of Edinburgh Systematic Image Review Service 2 (SIRS 2; https://sirs2.ccbs.ed.ac.uk/sirs2). Review of NCCT included presence, location of acute ischaemic lesions, presence of hyperdense vessels and background changes of brain frailty. For middle cerebral artery territory lesions, extent of early ischaemic change was graded using the Alberta Stroke Programme Early CT Score (ASPECTS).^16^ The presence and location of vessel occlusion was recorded for CTA review as per IST-3 protocol.^17^ We defined large vessel occlusion (LVO) as intracranial internal carotid artery (ICA), middle cerebral artery (MCA) M1 or basilar artery occlusion. Central CTP processing was undertaken using MiStar (v 3.2, Apollo Medical Imaging Technology, Melbourne, Australia) and documented volumes of ischaemic core (delay time > 3 s and relative cerebral blood flow (rCBF) <30%), penumbra (delay time >3 s and rCBF >30%) and core-penumbra mismatch.^18^ Target mismatch was defined as a perfusion lesion >15 ml and core:penumbra mismatch ratio >1.8, as used in the DEFUSE-2 study, the definition that had been shown to optimally identify patients with benefit from reperfusion at the time of the trial.^19^ For follow-up imaging, the presence and location of subacute ischaemic lesions, and the type of haemorrhage were recorded.

### Sample size and statistical analysis

We hypothesised that multimodal imaging would modify thrombolysis rates depending on imaging findings and clinical severity. We expected that patients with minor stroke symptoms would constitute the majority of patients and that in this group CTA or CTP abnormalities would increase the likelihood of thrombolytic treatment; secondly, we expected that a small proportion of patients would have large volumes of very severe CBF reduction (large core)^20,21^ and in this group the likelihood of thrombolytic treatment would be reduced; and finally, the remaining patients with moderately severe symptoms would likely be treated irrespective of CTA and CTP findings. We therefore anticipated a net increase in treatment numbers in the multimodal imaging group.

We assumed a thrombolysis treatment rate of 25%^22^ based on NCCT alone and estimated that 152 subjects per group would detect an increase in the proportion treated to 40% (absolute change of 15%) with 80% power at *p* = 0.05. Allowing for post-randomisation exclusion due to diagnosis of non-ischaemic stroke pathology based on NCCT in 15% of patients and allowing also for data acquisition and analysis problems yielding uninterpretable imaging of 10% in the multimodal imaging group, a total of 200 subjects per group was estimated as the sample size.

Continuous variables are summarised as mean and standard deviation (SD) if normally distributed, otherwise as median and interquartile range (IQR); categorical variables are summarised as frequencies and percentages. The proportions of patients treated with IV rtPA in the two groups were compared using logistic regression analysis adjusting for randomised group and minimisation variables. Adjusted Odds Ratios (aORs) for the effect of the imaging intervention are reported along with 95% confidence intervals (CI) and *p*-values. Exploratory analyses adjusted for baseline factors predictive of treatment with rtPA. Time to treatment decision was analysed using a stratified Wilcoxon rank sum test and time to treatment administration using a stratified log-rank test to account for censored outcomes. Analyses of the mRS used an ordinal shift analysis approach to seek evidence of differences between the two randomised groups in distributions across the entire scale of the mRS. aORs with 95% CIs and *p*-values are reported from ordinal logistic regression analysis adjusting for minimisation variables.

The primary endpoint was the proportion of patients receiving IV rtPA. Secondary endpoints were blinded 3 month functional outcome defined by the mRS distribution, the proportion with favourable outcome at 3 months defined by mRS of 0 or 1 (excellent outcome) and mRS 0–2 (independence); favourable early treatment response defined by an NIHSS score of 0–1 or improvement by ⩾8 points, at 24 h and at day 7; Safety outcomes were the proportion with Symptomatic Intracerebral Haemorrhage (SICH, defined as deterioration by ⩾4 points on the NIHSS and type 2 parenchymal haemorrhage (PH2) on follow-up brain imaging),^23^ and mortality.

Intervention effect differences between subgroups of the study population were assessed by including interaction terms within regression models. Subgroup effects were explored in relation to stroke severity, lacunar versus non-lacunar stroke syndrome and whether an infarct was visible on follow-up imaging. In all regression analyses, if models became unstable due to small numbers of events within some strata of the population, models were refitted with fewer adjustment variables.

No assumptions are made about missing data and all analyses use complete data only. All *p*-values are reported without adjustment for multiple comparisons. *p*-Values less than 0.05 are considered statistically significant. Analyses were conducted using R statistical software (R Core Team (2021). R: A language and environment for statistical computing. R Foundation for Statistical Computing, Vienna, Austria. URL https://www.R-project.org/), v4.0.4.

*Role of the Funding Source*: The trial was funded by the National Institute for Health Research (NIHR) Efficacy and Mechanism Evaluation Programme (Ref: 11/100/78). The funding body had no role in trial design, collection, analysis or interpretation of data, writing of the report or in the decision to submit for publication.

## Results

**Figure 1.**
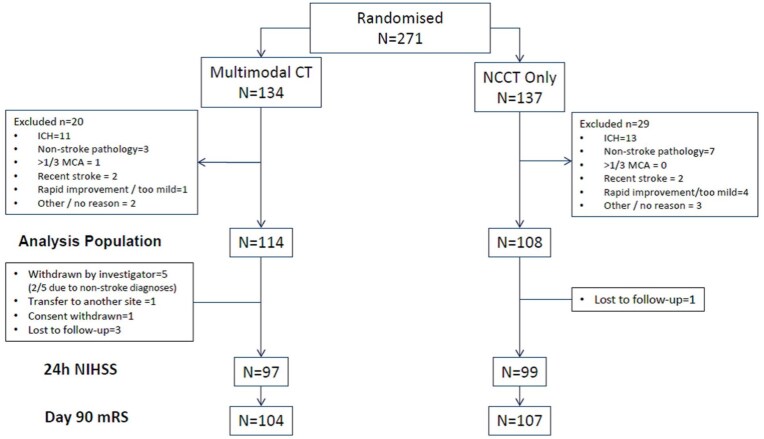
Flowchart showing screen failures and final analysis population. CT: computed tomography; NCCT: non-contrast computed tomography; ICH: intracerebral haemorrhage; MCA: middle cerebral artery; NIHSS: National Institutes of Health Stroke Scale; mRS: modified Rankin Scale.

Between March 2015 and May 2018, 271 of a planned 400 patients were randomised into the trial at 11 UK sites, 134 to multimodal CT and 137 to NCCT. Recruitment ceased when funding was unavailable to continue. After initial imaging 49 patients were excluded as ineligible for thrombolysis, mainly due to finding of intracerebral haemorrhage (*n* = 24) or non-stroke pathology (*n* = 10), leaving 114 thrombolysis-eligible patients in the multimodal CT group and 108 in the NCCT group (Figure 1).

Mean age was 67.5 years and 45.6% were female. Baseline characteristics of the population were well-balanced between multimodal CT and NCCT groups (Table 1). Median NIHSS score was 6 (interquartile range 3–12). Median onset to randomisation time was 122 min (interquartile range 90, 165). Time intervals did not differ between groups with respect to onset to randomisation or onset to first imaging (Table 2).

**Table 1.** Randomised population baseline characteristics and medical history. .

Characteristic	Multimodal CT	NCCT
*n*	114	108
Age, years, mean (SD)	66.9 (16.4)	68.3 (16.2)
Female, *n* (%)	61 (54.0%)	40 (37.4%)
NIHSS, median (IQR)	6 (3, 11)	6 (4, 12)
Stroke syndrome		
Lacunar syndrome	33 (28.9%)	18 (16.8%)
Cortical syndrome	65 (57.0%)	70 (65.4%)
Uncertain*	16 (14.0%)	19 (17.8%)
Estimated pre-stroke mRS		
0	65 (57.0%)	63 (58.3%)
1	28 (24.6%)	25 (23.1%)
⩾2	21 (18.5%)	20 (18.6%)
Underwent endovascular thrombectomy	2	1
Blood glucose (mmol/l), mean (SD)	6.9 (3.1)	6.9 (3.1)
Systolic blood pressure (mmHg), mean (SD)	149 (21)	153 (22)
Diastolic blood pressure (mmHg), mean (SD)	83 (14)	80 (14)
Medical history		
Myocardial infarction	15 (13.2%)	7 (6.5%)
Other ischaemic heart disease	11 (9.6%)	12 (11.2%)
Previous stroke	23 (20.2%)	24 (22.4%)
Diabetes	27 (23.7%)	13 (12.1%)
High blood pressure	62 (54.4%)	51 (47.7%)
Atrial fibrillation	10 (8.8%)	18 (16.8%)
Current smoking	14 (12.3%)	18 (16.8%)
Alcohol excess	3 (2.6%)	14 (13.1%)
NCCT findings		
ASPECTS median (IQR)	10 (9, 10)	10 (10, 10)
0–7	10 (8.8%)	8 (7.5%)
8–9	19 (16.7%)	12 (11.2%)
10	85 (74.6%)	87 (81.3%)
Hyperdense vessel sign	27 (23.7%)	16 (15.0%)
Established Brain Ischaemic Lesions	59 (51.8%)	70 (65.4%)
Brain atrophy	92 (80.7%)	94 (87.9%)
Extent of leukoaraiosis		
None	58 (50.9%)	66 (61.7%)
Low	0 (0.0%)	0 (0.0%)
Medium	31 (27.2%)	25 (23.4%)
High	24 (21.1%)	16 (15.0%)
CTA findings, *n*	96	17
Occluded intracranial vessel	21 (30.0%)	4 (44.4%)
CTP findings, *n*	99	0
*Core volume (ml) median (IQR)	6.5 (0.3, 14.2)	**-**
*Penumbra volume (ml) median (IQR)	25.8 (2.8, 66.3)	**-**
*Mismatch ratio	3.61 (2.65, 8.50)	**-**
*Target mismatch n (%)	7 (18.9%)	**-**
Follow-up brain imaging available modality	96 (84.2%)	97 (89.8%)
CT	69 (60.5%)	83 (76.9%)
MRI	27 (23.7%)	16 (14.8%)
Findings		
Confirmed infarct	47 (41.2%)	57 (52.8%)
Non-stroke pathology	0 (0.0%)	2 (1.9%)
No confirmed acute lesion	49 (43.0%)	38 (35.2%)

Imaging findings based on central imaging review.

NIHSS: National Institutes of Health Stroke Scale; mRS: modified Rankin Scale; SD: standard deviation; IQR: interquartile range; NCCT: non-contrast computed tomography; CTA: computed tomography angiography; CTP: computed tomography perfusion.

**Table 2.** Time intervals and imaging undertaken.

All times in minutes, median (IQR)	Multimodal CT	NCCT	*P*
Onset to randomisation	121 (88, 166)	122 (92, 157)	*p* = 0.921
Onset to NCCT	130 (95, 175)	123 (93, 162)	*p* = 0.448
CTA performed, *n* (%)	107 (93.9%)	18 (16.7%)	*p* < 0.0001
CTP performed, *n* (%)	103 (91.2%)	0	*p* < 0.0001
Onset to first multimodal scan (CTA or CTP) min, median (IQR)	130 (99, 184)	124 (109, 152),*	*p* = 0.795
Onset to treatment decision	160 (125, 207)	136 (113, 175)	*p* = 0.016
If rtPA given (*n* = 129)	131 (116, 180)	135 (108, 170)	*p*_interaction_ = 0.152
If not given (*n* = 93)	192 (141, 225)	150.0 (117, 185)	
Onset to rtPA administration	144 (120, 184)	145 (118, 174)	*p* = 0.697

NCCT: non-contrast computed tomography; CT: computed tomography; CTA: CT angiography; CTP: CT perfusion; rtPA; recombinant tissue plasminogen activator; IQR: interquartile range.

^*^Time to CTA for those allocated NCCT alone but who underwent additional CTA.

Of those randomised to multimodal imaging, 107 (94%) underwent CTA and 103 (91%) CTP; 90.3% of multimodal CT patients had both CTA and CTP. Of those randomised to routine imaging, 18 (17%) underwent CTA in addition to NCCT and none underwent CTP. No adverse events related to contrast administration were reported. CTA was deemed technically adequate by central review in 104/107 (97.2%) of multimodal CT patients and for all patients who underwent NCCT alone. CTP was successfully processed centrally in 93/99 studies available for central review (94%). Imaging findings are described in Table 1. There was substantial agreement between local and central assessment for the presence of any intracranial vessel occlusion (Kappa = 0.63, *p* < 0.0001) or large vessel occlusion (Kappa = 0.64, *p* < 0.0001). Of 35 CTPs locally interpreted as showing no perfusion abnormality, none had target mismatch on central review. Local site estimates for the extent of mismatch compared to centrally processed CTP showed fair to moderate levels of agreement (for >60% mismatch, kappa = 0.255, *p* = 0.012, and for ⩾20% mismatch kappa = 0.581, *p* < 0.001).

### Primary endpoint

Overall, 128 participants (57.7%) received thrombolysis. Significantly fewer multimodal CT patients received IV rtPA (56/114, 49.1%) compared to NCCT patients (73/108, 67.6%, adjusted Odds Ratio (aOR) 0.46 (95% CI: 0.25, 0.83), *p* = 0.0102).

### Secondary endpoints

Among those in whom a decision to treat with intravenous rtPA was made, onset to treatment decision time did not differ significantly by allocated imaging group (multimodal CT median 131 min (IQR 116, 180), NCCT median 135 min (IQR 108, 170), Table 2, *p* = 0.641).

**Figure 2.**
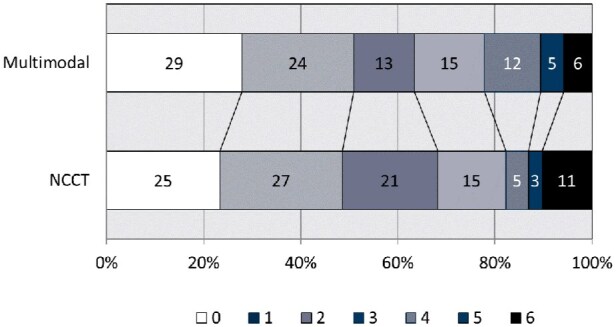
Modified Rankin scale distribution at day 90 follow-up. Adjusted odds ratio 0.93 (0.57, 1.51, *p* = 0.758). NCCT: non-contrast computed tomography; CT: computed tomography. Numbers in each section are the number of participants in each outcome category.

The proportional odds assumption for mRS distributions was met. The distribution of day 90 mRS functional outcomes did not differ by imaging group (Figure 2: aOR 0.93 (0.57, 1.51), *p* = 0.758). There were no differences in the proportions of patients achieving excellent (mRS 0–1) or independent (mRS 0–2) recoveries at day 90 (Table 3), nor in rates of major early neurological improvement (recovery to NIHSS score of 0 or 1, or an improvement of 8 or more points) at either 24 h (*p* = 0.913) or day 7 follow-up (*p* = 0.961).

**Table 3.** Primary and secondary outcomes.

Outcomes	Multimodal CT	NCCT	aOR (95% CI)	*p*
Primary outcome				
rtPA Given	56 (49.6%)	73 (68.9%)	0.38 (0.20, 0.71)	*p* = 0.0026
Secondary outcomes				
Onset to Treatment Decision (rtPA given)	131 min	135 min		*p* = 0.635
Onset to Treatment Time	144 min	145 min		
mRS Distribution	-	-	0.97 (0.58, 1.61)	*p* = 0.906
mRS 0–1	52 (52.5%)	50 (48.5%)	0.97 (0.53, 1.81)	*p* = 0.936
mRS 0–2	65 (65.7%)	71 (68.9%)	0.64 (0.33, 1.27)	*p* = 0.200
NIHSS 0–1 or ⩾8 point improvement at 24 h	48 (50.0%)	49 (50.5%)	0.98 (0.54, 1.76)	*p* = 0.935
PH1 ICH	2 (1.8%)	1 (0.9%)	1.91 (0.17, 21.38)	*p* = 0.599
PH2 ICH,*	0 (0.0%)	3 (2.8%)	-	
SICH,*	0 (0.0%)	2 (1.9%)	-	
Mortality	6 (5.3%)	11 (10.2%)	0.46 (0.16, 1.31)	*p* = 0.147

NCCT: non-contrast computed tomography; CT: computed tomography; rtPA: recombinant tissue plasminogen activator; mRS: modified Rankin Scale; NIHSS: National Institutes of Health Stroke Scale; PH1/2: parenchymal haematoma type ½; ICH: intracerebral haemorrhage; SICH: symptomatic intracerebral haemorrhage; aOR: adjusted odds ratio; CI: confidence interval.

^*^Odds ratios (and thus also significance) cannot be estimated for the two endpoints of PH2 and SICH since no events occured in one group.

Intracerebral haemorrhage (ICH) of any type was reported in 9/222 (4.1%) of patients, all of whom had final clinical diagnosis of confirmed stroke. This was graded as a parenchymal haematoma type 2 (PH2) in 3/222 (1.4%) and as symptomatic ICH in 2/222 (0.9%). No PH2 haematomas or SICH events occurred in the multimodal group, and all occurred in the NCCT group. Details of all PH1 and PH2 events are given in Supplemental Table 1. Death occurred in 6/114 (5.3%) assigned multimodal CT and 11/108 (10.2%) assigned NCCT (aOR 0.46 (95% CI: 0.16, 1.31), *p* = 0.147).

### Subgroup analyses

There was no interaction of thrombolysis decision by allocated imaging group in subgroups of stroke severity (NIHSS scores 0–6 vs 7–16 vs ⩾17, *p*_interaction_ = 0.147), stroke syndrome (lacunar or non-lacunar, *p*_interaction_ = 0.895), or whether follow-up imaging confirmed a new infarct (*p*_interaction_ = 0.452).

There was no interaction of treatment decision times with stroke severity by baseline NIHSS (*p*_interaction_ = 0.0618). Among those in whom a decision was made not to treat, onset to decision time was longer among those in the multimodal imaging group (192 (IQR 141, 225) min) than in the NCCT group (150 (IQR 117, 185) min) but there was no significant interaction between imaging group, treatment decision time and rtPA treatment (*p*_interaction_ = 0.152).

**Figure 3.**
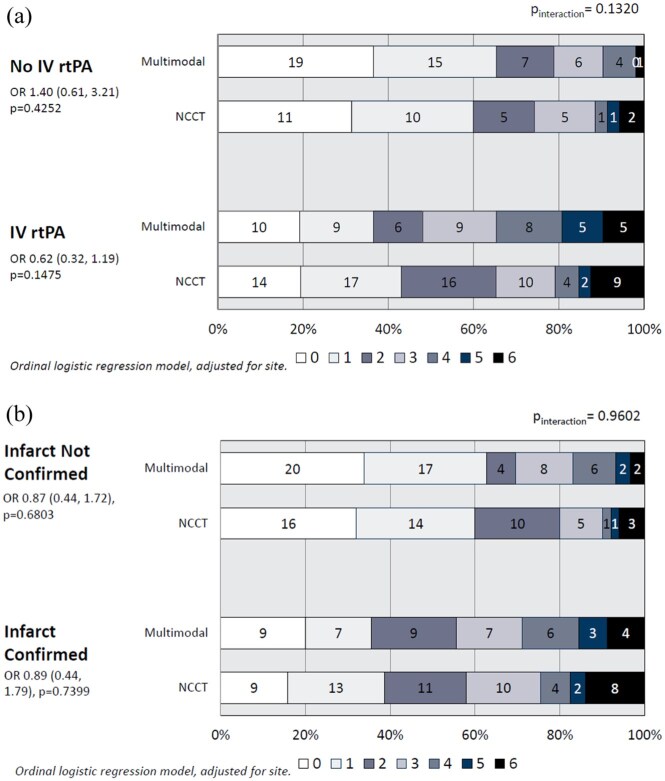
(a) Day 90 modified Rankin Scale distribution by whether thrombolytic therapy was delivered; and (b) by whether an infarct was visualised on follow-up imaging.

There were no interactions of day 90 mRS distribution with subgroups by NIHSS severity (*p*_interaction_ = 0.905), stroke subtype (lacunar vs non-lacunar or uncertain classification, *p*_interaction_ = 0.904), whether intravenous rtPA was given (Figure 3(a), *p*_interaction_ = 0.110), or whether an infarct was visualised on follow-up imaging (Figure 3(b), *p*_interaction_ = 0.960).

### Secondary analyses: imaging and thrombolysis decisions

The presence of early ischaemic change on NCCT was significantly associated with a decision to treat with IV rtPA in both groups (ASPECTS <10 vs 10, aOR for rtPA treatment in the multimodal CT group was 3.02 (95% CI 1.23, 7.41), *p* = 0.016, and for NCCT group 3.30 (0.90, 12.13), *p* = 0.073). In the multimodal CT group, the likelihood of giving rtPA was strongly associated with the presence of any intracranial occlusion on CTA (aOR 8.71 (95% CI 3.01, 25.21), *p* = 0.0001) or of a large vessel occlusion (aOR 7.69 (2.03, 29.21), *p* = 0.003). The presence of a CTP abnormality was strongly associated with administration of rtPA (for locally interpreted CTP, aOR 18.90 (6.85, 52.14), *p* < 0.0001 and for centrally processed CTP aOR 4.46 (1.72, 11.57), *p* = 0.002). Centrally processed CTP confirmed that no patient who was not given rtPA had a target mismatch.

Among 51 patients who underwent multimodal imaging and were not given thrombolysis, sites recorded their reason as absence of vessel occlusion in 35 cases (69%) or lack of acute ischaemia in 25 (49%), but large ischaemic core in only 2 (4%).

Follow-up imaging at 24h was available in 193 patients and included NCCT in 152 (79%) and MRI in 43 (22%). An infarct was confirmed on follow-up imaging in 104/222 patients (46.8%), 47/114 (41%) of those allocated multimodal imaging and 57/108 (53%) of those allocated NCCT. Among those allocated multimodal CT thrombolysis was given to 28/47 (60%) in whom an infarct was identified at follow-up and 28/67 (42%) with no infarct at follow-up; in those allocated NCCT thrombolysis was given to 46/57 (81%) and 27/51 (53%) respectively, *p*_interaction_ = 0.452. The likelihood of an infarct on follow-up imaging was related to greater clinical severity (NIHSS scores 7–16 vs 0–6, OR 1.76 (0.97, 3.18), *p* = 0.061, and for NIHSS scores >16 vs 0–6, OR 4.04 (1.65, 9.91), *p* = 0.002) and the presence of early ischaemic change on NCCT (ASPECTS <10, OR 6.58 (3.08, 14.08), *p* < 0.0001), but not to brain atrophy, presence of established ischaemic lesions or leukoaraiosis. Among those allocated multimodal imaging, an infarct confirmed on follow-up imaging was significantly associated with the presence of any perfusion abnormality, either locally (OR 3.44 (1.50, 7.90), *p* = 0.004) or centrally determined (OR 5.21 (1.94, 14.02), *p* = 0.001).

There was no significant difference between randomised groups in neurological deficit pattern based on NIHSS scores^24^ of those not thrombolysed (*p* = 0.468) or thrombolysed (*p* = 0.452). Patients not thrombolysed had lower total stroke severity (median NIHSS 4 in both groups) compared to those thrombolysed (median NIHSS 8 in the NCCT and 7 in the multimodal CT groups) and a preponderance of isolated symptoms and mild deficits.

## Discussion

Observational data have suggested better functional outcome and reduced mortality or SICH incidence with multimodal CT selection for thrombolytic treatment in acute stroke <4.5 h from onset,^25,26^ but this imaging strategy has not been investigated previously in a prospective randomised controlled trial. We found that use of multimodal CT reduced treatment rates with intravenous thrombolysis, with no evidence of detriment from lower rates of treatment with respect to day 90 functional outcomes or early neurological change and we observed numerically fewer SICHs and lower mortality among those allocated multimodal imaging, although neither outcome reached statistical significance. These findings concur with data from observational cohorts, registry data and subgroups of randomised trials.^13,25–27^ Additional imaging did not delay treatment initiation among those given thrombolytic treatment. Abnormal imaging findings with any modality were strongly associated with a greater likelihood of thrombolytic treatment, abnormal perfusion imaging being associated with the greatest odds of a decision to treat.

Treatment times among those thrombolysed were not significantly different in the multimodal imaging group, addressing one of the common concerns that door to needle times may be affected adversely by additional imaging. Reduced decision time appeared to offset additional scan acquisition and processing time.

In keeping with the evidence for endovascular thrombectomy among patients with intracranial LVO,^28^ CTA has become more widely used. In UK practice CTA is employed principally to screen for thrombectomy-eligible patients with significant clinical deficits (e.g. NIHSS ⩾ 6). CTP has been implemented to date primarily as a tool to identify patients who may benefit from reperfusion therapies when onset time is unknown, or presenting beyond 6 h, where trial evidence supports the value of perfusion-based imaging selection for reperfusion therapies.^29,30^ Our current findings indicate potential value of multimodal imaging within the first 4.5 h from symptom onset. Abnormalities on multimodal CT, particularly CTP, were strongly associated with increased likelihood of treatment and reflect the clinical value of positive diagnostic support for decision making in a time-critical environment where diagnosis otherwise relies on clinical assessment and non-contrast CT exclusion of haemorrhage. The association of treatment with presence of vessel occlusion on CTA may reflect a belief in vessel occlusion as a therapeutic target, but is part of a pattern suggesting that positive support from any diagnostic imaging modality modifies decisions.

Reduced thrombolytic use after multimodal imaging was the opposite effect from our original hypothesis. The higher than expected thrombolysis rate observed is consistent with selective randomisation of thrombolysis-eligible patients, and was higher than previously reported based on onset time).^5,22^ Our findings and other observational data suggest a shift in clinical behaviour towards a presumption in favour of treatment, perhaps encouraged by targets to increase treatment rates and minimise door-to-needle times.^6,31^ A notable concurrent development has been high reported rates of thrombolytic administration to patients subsequently diagnosed as having stroke mimics (13%–18% in recent clinical trials^7,8^). While observational data suggest a low incidence of serious complications of thrombolytic administration in mimics,^32^ SICH incidence of at least 1% has been estimated^32,33^ and thrombolytic delivery is associated with prolonged hospital stay and higher acute care costs.^34^ Stroke mimics may account for up to 31% of acute presentations to hospital services^35^ and 53% of all stroke assessments in hospitals in England and Wales in a national audit, in which they represented 1 in 13 of all patients thrombolysed.^9^ Mimic rates are near 50% among those with less severe symptoms.^36^ While a limitation of our data is that we did not collect data specifically on final diagnosis of stroke or stroke mimics, it is a reasonable assumption that mimics were recruited given the similarity of the trial population to that in clinical practice and recent acute stroke studies.

Health economic analysis was beyond the scope of the present study but would be of interest. Reduced (but potentially better targeted) use of thrombolytic agents may be cost saving, but additional imaging incurs costs for contrast and image processing software and uses more scanner time. There is wide variation in these costs across countries.

Local site interpretation of both CTA and CTP was consistent with central review, in particular for detection of LVO and CTP target mismatch. We defined core and penumbra thresholds optimised for MiStar software based on imaging outcome prediction.^18^ Volumetric thresholds for target mismatch were based on DEFUSE-2,^19^ which proposed criteria that signified optimal response to reperfusion and which were subsequently deployed as a selection criterion in trials of late-window reperfusion.^11^ The DEFUSE-2 mismatch definition had greatest evidence of clinical relevance at the time of the trial. Smaller degrees of mismatch such as a penumbra:core ratio of 1.2 may, however, appropriately select patients for thrombolysis or thrombectomy.^10^ Since post-processing software was not routinely available at study sites during this trial, visual assessment relied on comparison of maps produced by scanner software that did not routinely display threshold-based analyses. Even without automated threshold-based volume analysis, agreement on extent of mismatch was fair to moderate.

The presence of CTA or CTP abnormalities on imaging was strongly associated with the presence of a confirmed infarct on follow-up imaging. Those not treated had predominantly mild and limited neurological deficits. Given the limited spatial resolution of CTP and limited sensitivity of NCCT to small infarcts, it is likely that some patients with minor stroke were not treated. While we found no difference in functional outcomes at day 90, the trial had insufficient power to test non-inferiority of multimodal imaging and further investigation of functional outcomes in relation to imaging should be undertaken. It is possible that some patients who might have benefitted from thrombolysis did not receive treatment in the multimodal imaging group, which would be consistent with the non-significantly greater numbers with mRS 0–1 or 0–2 outcomes in the subgroups of those thrombolysed or with confirmed infarcts on follow-up imaging. Higher mRS 0–1 outcomes might also be a consequence of treatment of a higher number of stroke mimics. Considerable caution is required, however, since numbers in these subgroups are small and the trial was not designed to demonstrate differences in mRS outcomes. Nevertheless, our findings are consistent with data that question the balance of risk and benefit from thrombolysis among patients with minor stroke. Observational data have found thrombolysis to be associated with no difference in independent recovery but greater likelihood of both intracranial haemorrhage and very poor outcome (mRS 5–6) among patients with perfusion defects <15ml.^27^ Whilst treatment effect was not modified by NIHSS score in pooled analysis of randomised controlled trials of thrombolysis, those with low NIHSS represented only 10% of participants and were required to have a disabling neurological deficit.^1^ No heterogeneity of rtPA treatment effect was seen in the subgroup of patients in WAKE-UP with MRI-proven acute lacunar infarcts,^37^ but European guidelines consider evidence for treatment of lacunar stroke to be of very low quality.^38^ Recent evidence from TEMPO-2 indicates no benefit from thrombolytic therapy among patients with non-disabling neurological deficits^39^ even in the presence of imaging evidence of intracranial vessel occlusion.^40^ The PRISMS trial in patients with non-disabling stroke and NIHSS 0–5 found no significant difference in outcome between thrombolysis and control, but higher SICH incidence in the thrombolysis-treated group.^8^ Dual antiplatelet therapy was non-inferior to alteplase the ARAMIS trial in minor stroke.^39^

Reduced SICH incidence and mortality among patients meeting criteria for perfusion-based target mismatch has been reported in observational data,^25^ and is consistent with a lower incidence seen among patients undergoing thrombectomy who lack the target mismatch perfusion imaging profile.^19^ Potentially improved safety of thrombolysis by excluding minor stroke and stroke mimics is a plausible effect of greater imaging specificity guiding treatment decisions.

### Limitations

It is possible that clinicians were more likely to randomise patients in whom they were more uncertain about thrombolytic treatment and that the magnitude of the effect that we observed is not generalisable to more clinically certain diagnoses. However, the very high treatment rate in the NCCT group and similar population recruited compared to contemporaneous trials suggest that these data are relevant to clinical practice. The median severity of patients is representative of the hospitalised acute stroke population,^41^ and of recent clinical trials.^42^ The gradual introduction of endovascular thrombectomy during the recruitment period may have reduced recruitment of more severely affected patients, but intention to proceed directly to endovascular treatment was stated as a reason for not thrombolysing in only 7% of our patients. Imaging interpretation was reliant on local clinicians’ review of perfusion parameter maps obtained from the scanner, without additional processing to display predictive threshold-based volumetric data or artificial intelligence analyses, but was highly concordant with central analysis and did not miss any cases fulfilling target mismatch criteria, consistent with our prior findings on clinicians’ capability of interpreting CTP.^43^ We did not collect data on final clinical diagnosis at sites since this would likely be influenced by allocated trial imaging, and we did not mandate follow-up MRI that would have had greatest sensitivity for small infarcts. While our findings overall are consistent with reduced thrombolytic administration to stroke mimics, this interpretation is therefore speculative.

## Conclusions

In patients with clinically diagnosed acute ischaemic stroke being considered for possible thrombolytic drug treatment within 4.5 h of symptom onset, multimodal imaging reduced thrombolysis use. Time to decision making, time to treatment when thrombolysis was given and day 90 functional outcomes were not significantly different between groups, but these findings should be interpreted with caution since the trial lacked the statistical power to permit definitive conclusions. Lower incidence of SICH and mortality with multimodal CT, while not statistically significant, are consistent with previous observational data. Further investigation of functional outcome in a trial with appropriate sample size for these outcomes is merited. In addition to established uses in selecting patients for drug or device-based reperfusion beyond 6 h from onset or with uncertain onset time, multimodal CT may have value in aiding clinical decisions in patients within 4.5 h of onset.

## Supplementary Material

sj-docx-1-eso_23969873251372348

## Trial steering committee

Tom G. Robinson (chair), Phil White, Janet Freeman, Keith Muir, Elizabeth Warburton, Lalit Kalra, Joanna Wardlaw, Grant Mair, Ian Ford.

## Trial management

Alicia Murray.

## Independent data monitoring committee

Andrew Demchuk (chair), Jean Claude Baron, Alan Montgomery.

## Central imaging evaluation

Grant Mair, Joanna Wardlaw, Lesley Cala

## CTP analysis

Salwa El Tawil

## Participating sites, principal investigators and trial coordinators

Queen Elizabeth University Hospital, Glasgow (89 patients): KW Muir, S El Tawil, W Smith ; Queen Elizabeth Hospital Birmingham (53): J Khaira, J Cunningham; Countess of Chester Hospital (39): K Chatterjee, S Leason; King’s College Hospital, London (37): L Sztriha, B Piechowski, J Aeron-Thomas; Charing Cross Hospital, London (22): O Halse, P Wilding; William Harvey Hospital, East Kent (13): I Balogun, T Cosier; Glasgow Royal Infirmary (5): T Quinn, R Graham; Royal Stoke University Hospital (10): S Nayak, S Lyjko; Royal Preston Hospital (3): H Emsley, B Gregary; Salford Royal Hospital (1): C Smith, S Lee; Queen Elizabeth the Queen Mother Hospital, East Kent (1): G Gunathilagan, S Tilbey.

Study conception and design: KWM, IF, JMW, LK. Trial oversight and management: KWM, IF, JMW, GM, LK, TGR, PW, EAW, SET. Statistical analysis: IF, AM. Initial manuscript: KWM, IF, JMW, AM, SET. Data collection and review of manuscript: all authors.

All authors had full access to all study data and had final responsibility for the decision to submit for publication.

## Data sharing

Deidentified individual participant data and a data dictionary defining each field will be made available to others after planned publications are complete, and after approval of the proposed statistical analysis plan by the Trial Steering Committee and completed data sharing agreement.

## Trial registration

ClinicalTrials.gov identifier NCT02360670.

## Trial steering committee

Tom G. Robinson (chair), Phil White, Janet Freeman, Keith Muir, Elizabeth Warburton, Lalit Kalra, Joanna Wardlaw, Grant Mair, Ian Ford.

## Trial management

Alicia Murray

## Independent data monitoring committee

Andrew Demchuk (chair), Jean Claude Baron, Alan Montgomery.

## Central imaging evaluation

Grant Mair, Joanna Wardlaw, Lesley Cala.

## CTP analysis

Salwa El Tawil

## Participating sites, principal investigators and trial coordinators

Queen Elizabeth University Hospital, Glasgow (89 patients): KW Muir, S El Tawil, W Smith ; Queen Elizabeth Hospital Birmingham (53): J Khaira, J Cunningham; Countess of Chester Hospital (39): K Chatterjee, S Leason; King’s College Hospital, London (37): L Sztriha, B Piechowski, J Aeron-Thomas; Charing Cross Hospital, London (22): O Halse, P Wilding; William Harvey Hospital, East Kent (13): I Balogun, T Cosier; Glasgow Royal Infirmary (5): T Quinn, R Graham; Royal Stoke University Hospital (10): S Nayak, S Lyjko; Royal Preston Hospital (3): H Emsley, B Gregary; Salford Royal Hospital (1): C Smith, S Lee; Queen Elizabeth the Queen Mother Hospital, East Kent (1): G Gunathilagan, S Tilbey.

## Study registration

ClinicalTrials.gov identifier NCT02360670
