# Supplementary material for: Randomised, controlled Trial of CT perfusion and angiography compared to CT alone in thrombolysis-eligible acute ischaemic stroke patients: The penumbra and recanalisation acute computed tomography in ischaemic stroke evaluation (PRACTISE) trial
Source: Eur Stroke J. 2026 Jan 1;11(1):23969873251372348. doi: 10.1093/esj/23969873251372348 (PMC12866261; doi:10.1093/esj/23969873251372348)
Supplement: sj-docx-1-eso_23969873251372348 [file sj-docx-1-eso_23969873251372348.docx]

**Randomised, Controlled Trial of CT Perfusion and Angiography in Thrombolysis-eligible Acute Ischaemic Stroke Patients: the Penumbra and Recanalisation Acute Computed Tomography in Ischaemic Stroke Evaluation (PRACTISE) Trial**

**Online supplementary material**

**Detailed inclusion and exclusion criteria**

Inclusion Criteria

• Clinical diagnosis of stroke

• Written informed consent from patient, legal representative or consultee

• Male or non-pregnant female ≥18 years of age

• Within 4.5 hours of onset as defined by time since last known well

Exclusion Criteria

• Contraindications to thrombolytic drug treatment for stroke

• Pregnancy

• Known impaired renal function precluding CT contrast administration

• Known allergy to radiological contrast

• Severe concurrent medical condition that would prevent participation in study procedures or with life expectancy ≤ 3 months.

Contraindications to Intravenous Alteplase as per Summary of Medicinal Product Characteristics at the time of the trial:

- symptoms of ischaemic attack beginning more than 4.5 hours prior to infusion start or symptoms for which the onset time is unknown and could potentially be more than 4.5 hours ago
- minor neurological deficit or symptoms rapidly improving before start of infusion
- severe stroke as assessed clinically (e.g. NIHSS>25) and/or by appropriate imaging techniques
- seizure at onset of stroke
- evidence of intracranial haemorrhage (ICH) on CT scan
- symptoms suggestive of subarachnoid haemorrhage, even if CT scan is normal
- administration of heparin within the previous 48 hours and a thromboplastin time exceeding the laboratory upper limit of normal
- patients with any history of prior stroke and concomitant diabetes
- prior stroke within the last 3 months
- platelet count <100,000/mm^3^
- systolic blood pressure > 185 mm Hg or diastolic BP > 110 mm Hg, or aggressive management (intravenous pharmacotherapy) necessary to reduce BP to these limits
- blood glucose < 50 mg/dl or > 400 mg/dl (< 2.8mM or > 22.2mM)
- History of Increased bleeding risk:
  - significant bleeding disorder at present or within the past 6 months
  - known haemorrhagic diathesis
  - patients receiving effective oral anticoagulant treatment (e.g. warfarin sodium with INR > 1.3)
  - manifest or recent severe or dangerous bleeding
  - known history of or suspected intracranial haemorrhage
  - suspected subarachnoid haemorrhage or condition after subarachnoid haemorrhage from aneurysm
  - any history of central nervous system damage (i.e. neoplasm, aneurysm, intracranial or spinal surgery)
  - recent (<10 days) traumatic external heart massage, obstetrical delivery, recent puncture of a non-compressible blood-vessel (e.g. subclavian or jugular vein puncture)
  - severe uncontrolled arterial hypertension
  - bacterial endocarditis, pericarditis
  - acute pancreatitis
  - documented ulcerative gastrointestinal disease during the last 3 months, oesophageal varices, arterial-aneurysm, arterial/venous malformations
  - neoplasm with increased bleeding risk
  - severe liver disease, including hepatic failure, cirrhosis, portal hypertension (oesophageal varices) and active hepatitis
  - major surgery or significant trauma in past 3 months

Supplementary Fig SI. Treatment with intravenous rtPA by subgroups of stroke severity or stroke subtype.

Fig S2: Day 90 modified Rankin Scale distribution by subgroups of stroke severity.

Fig S3. Time to treatment delivery or decision by subgroups of stroke severity.

Table S1 Details of PH1, PH2 and SICH events.

| Case | Group | Age | Sex | NIHSS | ASPECTS | Prior Antithrombotic Therapy | Thrombolysis | ICH | Outcome | Narrative |
| --- | --- | --- | --- | --- | --- | --- | --- | --- | --- | --- |
| 1 | NCCT | 72 | Male | 12 | 7 | Aspirin | Yes | PH2, SICH | Died d5 | Improvement to NIHSS 8 after thrombolysis then deteriorated (NIHSS 15) |
| 2 | NCCT | 75 | Male | 17 | 10 | No | Yes | PH2, SICH | Died d2 | Hyperdense vessel, basilar artery occlusion on CTA |
| 3 | NCCT | 70 | Male | 10 | 10 | No | Yes | PH1 | mRS 1 |  |
| 4 | NCCT | 79 | Male | 20 | 10 | Clopidogrel | Yes | PH2 | mRS 4 |  |
| 5 | Multimodal | 49 | Male | 17 | 7 | No | Yes | PH1 | Alive at last follow up with NIHSS 3 | L MCA M1 occlusion on CTA |
| 6 | Multimodal | 83 | Male | 7 | 10 | Clopidogrel | Yes | PH1 | mRS 2 |  |
